# Vegetarian versus Meat-Based Diets for Companion Animals

**DOI:** 10.3390/ani6090057

**Published:** 2016-09-21

**Authors:** Andrew Knight, Madelaine Leitsberger

**Affiliations:** Centre for Animal Welfare, University of Winchester, Winchester SO22 4NR, UK; M.Leitsberger.15@unimail.winchester.ac.uk

**Keywords:** vegetarian, vegan, cat, dog, *Felis catus*, *Canis lupus familiaris*, *Canis familiaris*

## Abstract

**Simple Summary:**

Many owners of companion animals are interested in vegetarian diets for their animals, as concerns increase about the consequences of animal farming, for health, animal welfare, and the environment. However, are vegetarian diets for cats and dogs nutritionally balanced and healthy? This article comprehensively reviews the evidence published to date from four studies that have examined the nutritional adequacy of vegetarian diets for cats and dogs. To obtain additional information, we surveyed 12 pet food companies detailed in the most recent study. We also examined the nutritional soundness of meat-based companion-animal diets, and reviewed the evidence concerning the health status of vegetarian, carnivorous and omnivorous companion animals. Both cats and dogs may thrive on vegetarian diets, but these must be nutritionally complete and reasonably balanced. Owners should also regularly monitor urinary acidity, and should correct urinary alkalinisation through appropriate dietary additives, if necessary.

**Abstract:**

Companion animal owners are increasingly concerned about the links between degenerative health conditions, farm animal welfare problems, environmental degradation, fertilizers and herbicides, climate change, and causative factors; such as animal farming and the consumption of animal products. Accordingly, many owners are increasingly interested in vegetarian diets for themselves and their companion animals. However, are vegetarian canine and feline diets nutritious and safe? Four studies assessing the nutritional soundness of these diets were reviewed, and manufacturer responses to the most recent studies are provided. Additional reviewed studies examined the nutritional soundness of commercial meat-based diets and the health status of cats and dogs maintained on vegetarian and meat-based diets. Problems with all of these dietary choices have been documented, including nutritional inadequacies and health problems. However, a significant and growing body of population studies and case reports have indicated that cats and dogs maintained on vegetarian diets may be healthy—including those exercising at the highest levels—and, indeed, may experience a range of health benefits. Such diets must be nutritionally complete and reasonably balanced, however, and owners should regularly monitor urinary acidity and should correct urinary alkalinisation through appropriate dietary additives, if necessary.

## 1. Changing Patterns of Consumption

Major international, contemporary concerns; such as climate change, environmental degradation, public health, and animal welfare, are affecting and changing societal attitudes and activities.

Concerns about health follow increasingly widespread awareness of the links between the traditional “Western” diet, which is high in saturated fats, salt, and sugar, and low in fibre, and so-called “degenerative” diseases, such as cardiovascular disease, cancer, obesity and diabetes. The global transition from a predominantly plant-based diet to a diet high in animal products, as nations become increasingly affluent, has been identified as a noteworthy contributor to the rise in chronic disease [1,2].

Not surprisingly, the consumption of animals is even less beneficial for the animals themselves. By 2010, 66 billion terrestrial animals were being slaughtered annually [3]. This exceeded the entire human population almost by an order of magnitude. Trillions of fish are also believed to be killed annually. Modern high density, high throughput, and highly artificial systems, along with genetic selection for maximal productivity, and invasive husbandry procedures, have led to a range of serious animal welfare concerns in all major farmed animal species [4].

Major concerns also exist about the deleterious environmental impacts of animal agriculture. Climate change is rapidly developing into the biggest environmental issue for the current and subsequent generations. The industrialization of animal agriculture is an important contributor to global environmental degradation and climate change [5].

One important manifestation of such concerns relates to the diets of companion animals and their owners. In 2010, Leahy and colleagues [6] estimated that 1.5 billion vegetarians exist globally, of which 75 million are vegetarians by choice (compared to those in the developing world who are vegetarians by necessity, due to lack of choice). Vegetarians may be defined as those who consume foods derived from plants, with or without dairy products, eggs and/or honey (i.e., meat alone is excluded) [7]. Vegans are a subset of vegetarians, who eschew all consumption of animal products, including within cosmetics and household products. They also seek to minimise their use of products produced via animal testing. (Some vegans go so far as to avoid wines produced via traditional filtration using fish swim bladders. Vegan wines are available, which use alternate filtration methods [8]). In this article, the term vegetarian is routinely used, although many of the people and animals referred to are, in fact, vegan.

Leahy and colleagues [6] estimate that the sizeable global population of voluntary vegetarians will gradually grow, as education and affluence spreads.

### Pet Owner Concerns

Affluence is also positively associated with companion animal ownership. Driven by increasing concerns about health, the welfare of agricultural animals, and the environment, pet owners are increasingly interested in diets and lifestyles less consumptive of animal products [9].

Together with their animals, pet owners constitute a sizeable demographic group. With 95 million pet cats and 83 million pet dogs in the United States, cats and dogs combined outnumber the number of children under the age of 18 by almost two to one [10]. In the UK some 57 million pets are spread across 40% of households, and pet numbers have increased by one percent annually over each of the past five years [11]. Given the increasing proportion of consumers in developed nations who are concerned about health, animal welfare, and the environment—and accordingly interested in vegetarian diets for themselves and their families—it is not surprising that increasing numbers of consumers are also interested in the use of vegetarian diets for their companion animals [9]. Within the UK, three times as many vegetarian pet foods were launched in 2014 as in the previous three years [12].

In a recent study of 233 pet owners in Austria, Germany and Switzerland, 90% of participants said their convictions (moral, ethical, and animal welfare concerns) were the most important reasons that motivated them to maintain their pets on a vegan diet. Eighteen percent had to change their pet’s diet to a purely plant-based one, due to acceptance problems with ordinary feed [13]. This concurs with a study of the health status of 34 cats maintained on vegetarian diets, and 52 maintained on conventional diets, for at least one year, by Wakefield and colleagues (see the following) [14]. Of the 29 guardians of the vegetarian cats, 82% said they chose the diets due to ethical concerns. Similar results were found during a study of vegetarian dogs (86) and cats (8) in Germany, Switzerland, and Belgium [15].

Such concerns have also recently been voiced within the pet food industry [16], with some companies seeking alternative sources of protein, including fish, non-livestock terrestrial animals, and vegetables [17].

However, are vegetarian companion animal diets nutritious and safe? Some studies have indicated nutritional deficiencies in such diets. How do these compare with meat-based diets? Are vegetarian animals more, less or similarly healthy? Additionally, many feel vegetarian companion animal diets are not natural. How much of a concern is this for domesticated animal companions? Finally, for those pet owners that choose to feed vegetarian diets, how might they seek to maximise the health of their animal companions? All of these topics are explored in the following.

## 2. Biological Requirements of Companion Animal Diets

Both the domestic cat (*Felis catus*) and the domestic dog (*Canis lupus familiaris, Canis familiaris*) belong to the order of Carnivora. Their ancestral counterparts subsisted primarily or entirely on captured prey animals. Accordingly, they have a range of evolutionary adaptations designed to facilitate capture, apprehension, mastication, digestion and absorption of animal tissues. These include senses designed to detect prey animals, a musculoskeletal system designed to facilitate their capture, canine teeth to assist with apprehension, tooth crowns designed for cutting and slicing (rather than grinding of plant materials—which are more prominent in herbivores), and intestinal tracts that are shorter and have different digestive enzymes and intestinal flora than those of herbivores, which require relatively prolonged processing and digestion times.

However, perhaps as long as 33,000 years ago, dogs were domesticated from wolves [18,19]. These ancestral dogs were partially dependent on human food scraps. Accordingly, behavioural and physiological adaptations to a more varied diet—including plant-based foods—were necessary to allow ancestral dogs to thrive and achieve evolutionary success. Compared to carnivorous wolves, omnivorous dogs have evolved a superior ability to metabolize carbohydrates, and to subsist on a diet lower in protein [20]. Biochemical adaptations facilitating this include increased gene expression for pancreatic amylase, the ability to convert maltose to glucose, and increased intestinal glucose uptake [21]. Accordingly, the domestic dog is biologically omnivorous [13,21].

The cat, on the other hand, was domesticated roughly 10,000 years ago [22] and served a slightly different role. As well as being used as companions, cats were used to hunt animals deemed pests [23]. Even today, domesticated cats are well known for their predilection, in many cases, for continuing to hunt and kill wild animals. Accordingly, the selection pressures on cats to adapt to mixed human food scraps may have been less than those that applied to dogs, and they occurred over a significantly shorter duration. Unsurprisingly, therefore, cats generally lack the genetic, biochemical and behavioural adaptations that enable dogs to thrive on an omnivorous diet, and indeed, domesticated cats select a macronutrient profile (52% of metabolisable energy (ME) from protein) similar to the diet of wild cats [20].

However, interesting though they may be, it is important to acknowledge the limited relevance of such evolutionary adaptations to the modern, domesticated lifestyles of cats and dogs who are fed commercial diets from cans or packets at predictable times daily. Natural selection favoured the selection of attributes that enabled ancestral dogs and cats to survive long enough to reproduce within their historical environments. Those attributes (e.g., preference for calorie-dense foods, a desire to hunt, and unrestrained feeding behaviour) are not necessarily well suited to modern, domesticated environments and prolonged lifespans, where they may result in undesirable behaviours or health problems, such as obesity and its sequelae [24].

In order for dogs and cats to thrive within modern, domesticated environments—for the duration of their artificially increased lifespans—they must be provided with diets that are sufficiently palatable, bioavailable, and that are nutritionally complete and reasonably balanced. Each species and life stage (e.g., juvenile, adult, pregnant, lactating, geriatric) requires a particular nutrient profile; the provision of which can prevent malnutrition and can assist in the management of other diseases.

Although special attention must be paid to ensure adequate levels of certain nutrients such as proteins, amino acids (e.g., taurine, carnitine, methionine, lysine, and tryptophan), vitamins (e.g., Vitamins A, B3, B9, and B12), minerals (e.g., calcium, iron, zinc, and copper), and certain fats [25], it is important to remember that dogs, cats—and indeed all species—require specific nutrients, rather than specific ingredients. There is—at least in theory—no reason why diets comprised entirely of plants, minerals, and synthetically-based ingredients (i.e., vegan diets) cannot meet the necessary palatability, bioavailability, and nutritional requirements of cats and dogs [25]. Indeed, a growing number of commercially-available diets [26] aim to do so.

Such diets are supplied completely, or as supplements to be added to (sometimes cheaper) homemade diets, for which recipes may be supplied. Such recipes are also available in books (e.g., *Vegetarian Cats and Dogs* [27]). However, the homemade option appears to be less popular. In a study of European users of these diets, Semp [13] found that 39% of participating dog and cat owners used only complete diets; 9% used only homemade diets; while the remaining 52% primarily used complete diets, with regular addition of some homemade ingredients.

## 3. Nutritional Adequacy of Diets

The National Research Council (NRC) of the U.S. National Academy of Sciences is the leading provider of nutrient recommendations for dogs and cats. These form the basis for the Association of American Feed Control Official’s (AAFCO) nutrient profiles. AAFCO, in turn, provides mechanisms for developing and implementing uniform laws, regulations, standards and enforcement policies pertaining to such diets [28].

Other countries and regions have frequently developed their own nutritional guidelines based on country-specific legislation (e.g., Regulation (EC) No 767/2009 for Europe [29]) and with reference to AAFCO guidelines (e.g., the European Pet Food Industry (FEDIAF) on feeding trials [30,31]; the Working Group on the Labelling and Advertising of Pet Food in Canada on ingredient labelling [32]). The World Small Animal Veterinary Association (WSAVA) similarly provides global nutritional assessment guidelines [33].

Companion animal diets may be accompanied by labelling claims that they are nutritionally complete and balanced. There are two recognised methods of substantiating such claims, and within the U.S., the label’s nutritional adequacy statement must specify the method used [34]. The first method is to formulate the diet to meet AAFCO Dog and Cat Food Nutrient Profiles. The second method (which is considered the gold standard) [35], is to conduct a feeding trial for the specified life stage, using AAFCO-approved protocols. Upon successful completion of an appropriate feeding trial, the pet food is exempt from requirements to formulate it to meet nutrient profiles [36]. If nutritional claims cannot be substantiated using either method, the product must be clearly labelled as a snack, treat, or dietary supplement, or must contain a statement that indicates it is “intended for intermittent or supplemental feeding only” [34].

Such standards are intended to ensure that companion animal diets are nutritionally sound and that their nutritional content is accurately understood by consumers. However, a number of studies have raised concerns about the nutritional adequacy of vegetarian or vegan companion animal diets.

### 3.1. Study by Kanakubo et al. (2015) [37]

Most recently, Kanakubo and colleagues examined 13 dry and 11 canned vegetarian diets for dogs and cats that were sold in all or most of the United States. Crude protein (CP) and amino acid (AA) concentrations were compared with AAFCO Dog and Cat Food Nutrient Profiles. Minimum CP concentrations for the specified species and life stages were met by 23 diets; the remaining diet passed appropriate AAFCO feeding trials. However, 25% (6/24) diets did not meet all AA minimum requirements.

### 3.2. Company Responses

Twelve companies based in the U.S. and internationally supplied the 24 diets examined. In late 2015, we contacted all of these companies by email. We inquired whether they had any additional data or information relevant to the conclusions of Kanakubo and colleagues [37]. In particular, we asked whether companies could supply any evidence (e.g., studies by external independent laboratories or internal assessments) to verify the nutritional adequacy of their products. We also inquired about any steps taken during the manufacturing process to ensure that diets are nutritionally adequate, and consistent over time.

An initial email inquiry was followed by an additional email to non-responders after one month. Five companies (Ami, Evolution Diet Pet Food, Purely for Pets, Big Heart Pet Brands, Central Garden and Petco) failed to respond to either inquiry. An additional company (Natural Balance Pet Foods) declined to provide comments. Responses from the remaining six companies varied substantially.

Some of these six asserted that their products were nutritionally adequate. Royal Canine, for example, asserted that, “Canine vegetarian dry and wet diets are formulated to meet the nutritional levels established by the AAFCO Dog Food Nutrient Profiles for maintenance”.

Similarly, Purina Nestle asserted that, “The nutritional adequacy of our products is fundamental. We ensure this through formulation, feeding trials, and strict manufacturing processes. We meet or exceed every major food quality and safety standard, including those issued by the FDA, USDA, AAFCO and FEDIAF”.

V-dog asserted that their diet is “formulated to meet or exceed the nutritional levels established by the AAFCO Dog Food Nutrient Profiles for adult maintenance”. They even supplied the ingredient lists for their products and a nutritional breakdown. They also asserted that “Our production facility has staffed animal nutrition experts and veterinarians that conduct regular tests on the kibble post production to ensure its nutritional adequacy. We obtained our kibble’s guaranteed analysis from an external lab and our regular nutrition and quality tests are performed by our production facility on a regular basis”. They stated that they had not performed feeding trials, however “we’ve been in business for over 11 years and have seen thousands of dogs thrive into their golden years on the v-dog kibble”.

PetGuard asserted that, “In addition to independent laboratory tests for determining the level of vitamins, minerals and nutrients in our finished diets, we conduct home feeding studies to determine the performance of our diets in an actual home environment. It is an ongoing program for our dog and cat foods. The only compensation these pet parents receive for completing health and behavioural forms six times per year—and submitting the yearly veterinarian check-up report—is the foods themselves. Canned, kibble, treats or snacks, all are evaluated. We have done this for the past 20 years and are confident and pleased with our data that shows our diets perform, as a single source diet should, for the long term health and long life of our study group’s companions (average 16 years for canines and 18 years for felines)”.

No company provided details of independent laboratory verification of the nutritional content of their diets. PetGuard also stated that, “Our data is proprietary and we will not be able to share it with you. In a very competitive environment—where innovation and creativity comes from hard work, study, and technical manufacturing breakthroughs—discretion is necessary”.

Clearly, few companies asserted that they had used feeding trials or independent laboratory verification of nutritional composition. Evanger’s Dog and Cat Food Company provided some insights into the possible reasons for this: “Many brands send their foods to third-party laboratories to make sure they meet their guaranteed analyses, which typically doesn’t include the complete AAFCO breakdown as that would cost a few thousand dollars more—and no one makes that in profit on a batch of food. This is why manufacturers rely on help from their vitamin and mineral premix suppliers for help when formulated (*sic*) the food. They would assist in the inclusion rates and the knowledgeable ones would even provide degradation results for the age and processing of certain vitamins”.

In one case (Wysong), the company admitted that its product was unlikely to be nutritionally complete: “Wysong does not advocate the singular feeding of VeganTM to carnivores such as dogs and cats. … It is designed for intermittent feeding or as a base to add different meats for sensitivities and allergies”. They further asserted their philosophical opposition to the concept of attempting to produce a single, nutritionally complete diet: “Complete knowledge of nutrition does not exist … and therefore “completeness” is misleading. … With that in mind, Wysong seeks to formulate and educate for optimal, not “adequate” nutrition and encourages pet owners to rotate, vary, and enhance the diet”.

### 3.3. Author Conclusions

Kanakubo and colleagues [37] expressed concerns regarding the suitability of the diets examined and concluded that veterinary therapeutic diets might be more suitable because they met nutritional and labelling requirements. All three veterinary diets assessed met nutritional adequacy and labelling requirements, compared to only five of 21 over-the-counter diets that met both nutritional adequacy and labelling requirements. As Verma [24] commented, with respect to these results, “All three of the diets are produced by companies that are hailed by many veterinary nutritionists for their quality control measures, even more so for their prescription lines”. However, Verma also noted some concerns for owners preferring vegan diets: “None of the three current veterinary diets are completely free of animal-derived nutrients”.

### 3.4. Study Limitations

As acknowledged by Kanakubo and colleagues, their study included certain limitations that precluded definitive conclusions about the nutritional adequacy of vegetarian companion-animal diets. These included the small sample size (13 dry and 11 canned vegetarian diets for dogs and cats), the collection of dietary samples at only one point in time, from only one batch of each product, and possible variation due to methods used in particular laboratories (analytical variation). Although Kanakubo and colleagues reported low assay variability for both AA and CP analyses, substantial variations in results attributable to laboratory methods are possible, and indeed are allowed for within AAFCO guidelines [38].

Also significant was the lack of assessment of animals maintained on these diets (only one of which had previously passed a feeding trial). Blood concentrations of nutrients are affected by bioavailability and are more biologically relevant than dietary nutritional analyses; and concentrations of AAs are arguably more important than CP concentration *per se*. Studies in other species indicate that the sum of essential AA concentrations is not necessarily correlated with CP concentration, and that neither are necessarily correlated with biological indicators of CP adequacy—such as weight gain [39]. Accordingly, for the most reliable assessment of dietary adequacy, blood concentrations of essential AAs and CPs should be combined with the assessment of biological indicators of AA and CP uptake and utilisation—i.e., with clinical assessments.

### 3.5. Study by Semp (2014) [13]

Some older studies have also raised concerns about the nutritional adequacy of vegetarian or vegan companion animal diets. Semp [13] examined four canine and two feline vegan diets used by 233 pet owners from Austria, Germany, and Switzerland, along with Benevo Duo—a wet feed that aims to fulfil most nutritional requirements for both cats and dogs. Results were reported within a veterinary medical university thesis. Diets were analysed (at the University of Veterinary Medicine in Vienna, and Futtermittel-Labor Rosenau (Rosenau Feed Laboratory)) and compared with company feed analyses where available.

Diets were occasionally found to be deficient in energy (three diets), calorie (three diets) protein (one diet), and in potassium (three diets). The recipe for a homemade canine diet mostly fulfilled nutrient requirements, but was too low in the amino acids methionine and cysteine, vitamin B12, and sodium. Semp reported that these diets mostly fulfilled nutritional requirements and that no clinical abnormalities were associated with them (See the following).

### 3.6. Study by Gray et al. (2004) [40]

These investigators subjected two commercially-available vegan cat foods to blind nutritional analyses by an independent laboratory. Vegecat KibbleMix (a supplement designed to be added to a homemade diet) was prepared according to company instructions. Evolution canned diet for adult cats was marketed as a complete diet, requiring no additional preparation. The study showed both brands to be deficient in taurine, methionine, and arachidonic acid; with the Vegecat KibbleMix diet also deficient in Lysine and Arginine. The Evolution diet was deficient in several B vitamins, as well as retinol, calcium, phosphorus, and overall protein.

Concerned by these results, one of us (AK) contacted the manufacturers in 2005. Based on their responses, the most likely explanation was that both of the samples tested were nutritionally inadequate, but that most samples sold and used are adequate, and that formulation errors occurred at these factories [41].

Gray and colleagues only examined a single sample of each diet, limiting the broader applicability of their results. They noted: “(We) agree that our results do not prove that all vegan cat foods are nutritionally inadequate or that cats are incapable of surviving without meat. We looked at only two foods, so our conclusions are appropriate only for the foods analysed. We recognized study limitations in terms of the number of samples analysed and acknowledged that variations among batches or in nutrient content of key ingredients could explain our results.”

Given that this study is over a decade old, its relevance to current production processes must be questioned. Indeed, it is reasonable to hope that manufacturing and quality control standards may have improved over such a significant period of time.

### 3.7. Study by Kienzle and Engelhard (2001) [15]

These investigators studied 86 vegetarian dogs and eight vegetarian cats in Germany, Switzerland, and Belgium. Among the twelve prepared complete vegetarian dog foods investigated, the authors were able to recommend only two without reservation. Common formulation errors included insufficient protein, insufficient calcium (Ca), phosphorous (P), and an unbalanced Ca:P ratio; insufficient sodium, Vitamin A, Vitamin B12, taurine, and arachidonic acid; and trace element deficiencies. Additionally, although the feline diets investigated contained taurine, in all cases this was inadequate.

However, this study is now around 15 years old, and it is hoped that manufacturing standards and quality control processes may have since improved in the cases of these diets.

## 4. Consistency and Adequacy of Pet Food Diets Generally

Concerns about the nutritional adequacy of commercially available companion animal diets are not limited to vegetarian products. This was aptly demonstrated by Hill and colleagues in 2009 [42]. They utilized the “sample check program” offered by certain U.S. states, to check the level of concordance between labelling guarantees of nutritional content, and actual nutritional contents—as measured via laboratory analysis. Reports were analysed from five states (South Dakota, Indiana, New York, New Jersey, and Rhode Island) that were willing to provide these data free of charge. These included guaranteed and measured nutrient analyses of 2208 pet foods manufactured by 204 companies. Included were 1158 canned foods, 750 dry foods, 258 treats, 32 other types of foods (soft-moist, soft-dry, liquid, supplemental, or foods in pouches), and 21 foods of unidentified types.

In most cases, variations between labelling claims and measured values were low. (Mean differences were 1.5% for crude protein, 1.0% for crude fat, −0.7% for crude fibre, −4.0% for moisture, and −0.5% for ash). However, Hill and colleagues also noted that, “Within all types of food, there were a few outlying values where actual food analyses diverged markedly from the guarantee. For such foods, the adjusted estimate of composition would remain wildly inaccurate”.

They also noted that, “In absolute terms, the mean differences in analyses were mostly small. However, as a percentage of the amounts of each nutrient in the diet, the mean changes were substantial (5%–30%). Such inaccuracy can substantially affect any estimate of the ME density of the food obtained by calculation”. They noted that metabolisable energy (ME) density increases were substantial, when using either of two calculation methods recommended by the National Research Council. Such errors could partly explain increasing and concerning rates of obesity and a range of sequelae in modern companion animals [43].

The study by Hill and colleagues was not without limitations of its own. They noted that, “The choice of State laboratories was a sample only, and the choice of foods was decided by the State laboratories. It is possible, therefore, that the foods chosen may not be representative of all foods sold in the United States, and a more extensive body of data obtained more systematically might provide slightly different results”.

In short, although concerns exist about the nutritional adequacy of vegetarian companion animal diets, these concerns are not limited to such diets, and include a wide range of meat-based companion animal food products. Indeed, it is highly plausible that repeated independent laboratory analyses of a range of commercial products, vegetarian or meat-based, would similarly demonstrate nutritional inadequacies and inconsistency of nutritional content over time.

Of course, such findings in no way negate the ability of well-formulated vegetarian or meat-based diets to meet all the nutritional requirements of the normal animals for whom they are intended; they merely illustrate the need for good quality control during production.

As noted previously, following our 2015 survey of 12 U.S. pet food companies, Evanger’s Dog and Cat Food Company stated that, “Many brands send their foods to a third-party laboratory to make sure they meet their guaranteed analysis…” As Gray et al. [44] noted, “We consider it the responsibility of all pet food manufacturers to submit samples of their diets from multiple lots for independent nutritional analysis before claiming adequacy as a sole source of nutrition for cats or other species”.

## 5. Health of Vegetarian Companion Animals

Information about the nutritional adequacy of vegetarian brands and the bioavailability of their ingredients is very important. However, when considering the effectiveness of a diet, assessing the health of animals maintained on them carries even greater weight. This is why feeding trials—which assess the health of animals maintained exclusively on test diets, over time—are considered the gold standard when assessing dietary adequacy [35].

A growing body of evidence appears to indicate that dogs and cats can survive, and indeed thrive, on nutritionally-sound vegetarian and vegan diets. Numerous cases are described on various websites [45,46] and in a small number of books [27]. Benefits commonly reported, after transitioning dogs and cats to nutritionally sound vegan or vegetarian companion animal diets, include: decreased ectoparasites (fleas, ticks, lice and mites) and food intolerance reactions; improved coat condition; obesity reduction; regression in signs of arthritis; diabetes; cataracts; urogenital disease; and improved vitality.

However, although such case reports may be useful in suggesting areas on which to focus more rigorous research efforts, they do not (in themselves) meet the scientific standard of proof. To achieve this, well-designed, randomized controlled trials (RCTs) are needed [47]. Systematic reviews of multiple RCTs, ideally with meta-analyses of pooled results, provide the most reliable evidence [48,49].

However, maintaining dogs on unvarying diets within laboratory settings for prolonged periods can raise ethical concerns, and such studies—which could supply the highest standards of evidence within this field—are lacking. Nevertheless, a growing body of controlled feeding trials and population studies does exist, which shed light on the health of companion animals maintained on vegetarian diets long-term.

### 5.1. Study by Semp (2014) [13]

Semp studied companion animals in Austria, Germany and Switzerland. Owners were sourced using notice boards in veterinary practices, articles in various Facebook forums, and word of mouth. They were asked to complete a questionnaire about their experience feeding their cats and dogs a vegan diet, which was followed by a clinical examination and blood tests on 20 dogs and 15 cats that were randomly selected. The standardized clinical examination included assessments of general appearance, body condition, skin and coat, lymph nodes, vital signs; cardiovascular, respiratory and digestive systems; and defecation. Haematological (complete blood count) and biochemical (liver, kidney, and pancreatic) parameters were assessed, as well as levels of magnesium, calcium, iron, total protein, folic acid, vitamin B12, and carnitine.

### 5.2. Results

Two hundred thirty-three pet owners completed the questionnaire, including 174 dog and 59 cat owners, some of whom had both species. Animal participants were required to have eaten an exclusively vegan diet for at least six months; and to ensure dietary integrity, cats were required to have lived indoors only. Participating dogs had eaten vegan diets for six months to seven years, with a mean of 2.83 years. Participating cats had eaten vegan diets for six months to 6.5 years, with a mean of 3.9 years.

Thirty-nine percent of participating owners used only commercially-available diets. Nine percent used only homemade diets, and the remaining 52% used mostly commercially-available diets (but regularly mixed these with homemade ingredients).

Thirty-eight pet owners independently reported healthier and shinier coats after transitioning to vegan diets. Some animals, previously prone to scaly or oily coats, no longer showed signs of dermatological problems. Sixteen owners described improved odours of their pets. Some also noted increased stool volumes and improvement of stool consistency.

During standardized clinical examinations, no abnormalities were detected that were associated with diet. Most examined dogs and cats appeared happy and bright, although a minority were fearful or aggressive—reflecting the normal domesticated populations.

When considering blood test results, serum total protein of all 20 dogs and 15 cats studied were within normal ranges. For the dogs, no significant differences were evident in any of the tested parameters, compared to the dogs fed a conventional diet. In particular, lower levels of iron and vitamin B12 in vegan dogs were not observed. Not even the 10% (2/20) dogs fed a homemade supplemented diet showed any significant deviations.

For the cats, the main abnormality observed was significantly lower folic acid values (*p* < 0.001) in vegan cats, compared to conventionally fed cats. Semp stated that, “The reason … is not known and may need further investigation”. In cats, folate deficiency is associated with hyperhomocysteinemia [50], which may be associated with thromboembolic disease, although this is not described as an important risk factor [51].

No other significant deviations from normal values were observed. In particular, lower values of iron, protein or vitamin B12 in vegan cats were not observed.

### 5.3. Study by Brown et al. (2009) [52]

It is difficult to envision any companion animals placed under greater physical demands than sprint-racing Siberian Huskies. During sprint races, these dogs run fast through snow, while hauling sleds, for much of the 30-mile race duration [53].

In 2009, Brown and colleagues [52] reported the results of a study of 12 sprint-racing Siberian Huskies fed either a commercial diet recommended for active dogs (*n* = 6), or a meat-free diet formulated to the same nutrient specifications (*n* = 6). The commercial diet contained 43% poultry meal, which was replaced by maize gluten and soybean meal in the meat-free diet. The dogs were fed these diets for 16 weeks, which included 10 weeks of competitive racing.

Health checks were conducted by a veterinarian blinded to the dietary regimens. All dogs were assessed as being in excellent physical condition, and none developed anaemia or other detectable health problems.

### 5.4. Study by Wakefield et al. (2006) [14]

In 2006, Wakefield and colleagues published the first study of the health of a population of cats maintained on vegetarian diets (most, in fact, were vegan), long-term. Thirty four were maintained on vegetarian diets and 52 on conventional diets, for at least one year. No significant differences existed between the two groups in age, sex, body condition, housing, or perceived health status. Most of the caregivers in both groups described their cats as healthy or generally healthy.

Wakefield and colleagues also measured blood taurine and cobalamin (Vitamin B12) levels of 17 of these cats that had exclusively been fed either a commercial or homemade vegetarian diet. Cobalamin levels were within the normal range in all cases, and taurine levels were similarly normal in 82.4% (14/17) of cases. The remaining three cases were cats who were partly maintained on dinner table scraps. Because such scraps are not nutritionally complete or balanced, these should always comprise a minority of diets.

### 5.5. Study by Kienzle and Engelhard (2001) [15]

During a study of 86 vegetarian dogs and eight vegetarian cats in Germany, Switzerland, and Belgium, Kienzle and Engelhard [15] found numerous dietary deficiencies (see previous). Surprisingly perhaps, no clinical problems were found in the adult dogs. However, one cat showed symptoms of retina atrophy, and two displayed reduced frequency of oestrus.

### 5.6. Study by PETA (1994) [54]

In 1994, People for the Ethical Treatment of Animals (PETA) reported results of a systematic survey of the health of 300 vegetarian dogs sourced from 33 states within the U.S. and Canada via PETA’s newsletter [54,55]. Dogs ranged in age from young puppies to 19 years old, and included a wide range of breeds, males and females, both neutered and entire. Of these, 65.3% (196/300) were vegan, with the remaining 34.7% (104/300) simply vegetarian. They had been maintained on these diets for anywhere from less than two, to over nine years, with an average of 5.7 years. The precise diets used, and their level of nutritional adequacy, are unknown.

**Figure 1:**
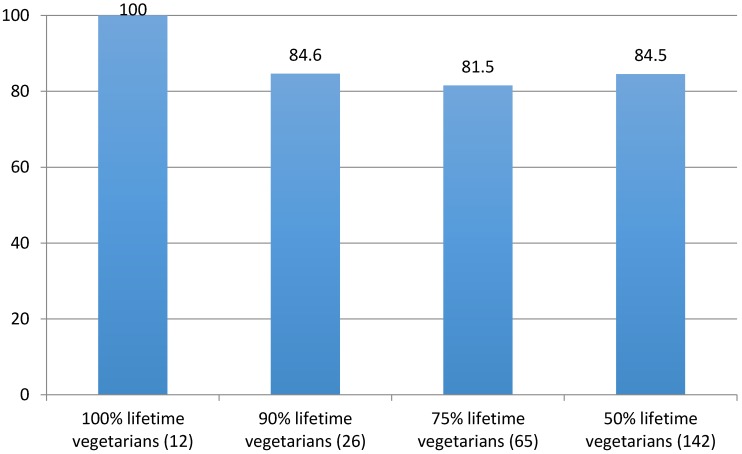
Percentages of dogs in good to excellent health, vs. time as vegetarians (PETA 1994) [53].

Over 80% of dogs maintained on vegan or vegetarian diets for 50% to 100% of their lifetimes were reported as being in good to excellent health (Figure 1).

Twenty-eight deceased dogs were included in the survey, with the median age of death being 12.6 years. The most common causes of death were cancer (eight) and heart disease (seven).

The most common health problems were infections of several kinds. Less common problems included excessive body weight (apparently, with no adverse clinical or behavioural effects), digestive problems (especially in older animals), hypothyroidism, vision and hearing deficits. Full details have been reported elsewhere [55]. All of these problems are also commonly reported within the normal domesticated dog population.

### 5.7. Study by Leon et al. (1992) [56]

This old study by Leon and colleagues confirmed that cats maintained on nutritionally-deficient diets may experience health problems. In this case, the vegetarian diet was formulated to be deficient in potassium, and clinical signs involved neuromuscular function.

### 5.8. Summary

The standard of evidence offered by these studies and case reports varies significantly, and very few, if any, meet the standards of well-designed RCTs that are considered the cornerstone of Evidence-Based Medicine [47]. Additionally, blood tests are rarely comprehensive. Accordingly, caution must be exercised before drawing definitive conclusions from these results.

Nevertheless, a significant and growing body of population studies and cases suggest that cats and dogs may be successfully maintained on nutritionally sound vegetarian diets long-term, and indeed, may thrive. Such diets have been associated with benefits such as improved coat condition, allergy control, weight control, increased overall health and vitality, arthritis regression, diabetes regression, cataract resolution, and decreased incidences of cancer, infections, hypothyroidism and ectoparasites (fleas, ticks, lice and mites). Deviations from normal ranges within blood test results do occur, but are uncommon, and rarely appear associated with clinical signs of disease.

Of course cats and dogs maintained on these diets also experience health problems, and occasionally die. Diseases such as cancer, heart disease and infections do occur. However, such diseases are also prevalent within the normal domesticated companion animal population.

## 6. Health of Omnivorous and Carnivorous Companion Animals

### 6.1. Meat-Based Diets

When considering dietary choices for companion animals, the hazards of meat-based diets, and any health problems experienced by animals maintained on them, should be considered—along with those experienced by vegetarian animals. Meat-based companion animal diets are not without significant hazards of their own.

These include contamination with *Salmonella*, *Listeria*, and a range of other potentially pathogenic microorganisms [57]—some of which may also cause disease in human cohabitants (particularly, where immunosuppressed); the prion proteins which cause transmissible spongiform encephalopathies, such as bovine and feline spongiform encephalitis; and mycotoxins (fungal toxins)—notably aflatoxins produced by *Aspergillus flavus* and *A. parasiticus*, and vomitoxin produced by *Fusarium* moulds [58].

Occasionally, very serious problems occur, leading to major recalls of pet food brands. Within the U.S., 11 such recalls occurred between 1996 and 2010. These resulted from chemical contaminants or misformulations: three aflatoxin, three excess Vitamin D3, one excess methionine, three inadequate thiamine, and one adulteration with melamine and related compounds. In addition, there were two additional warnings concerning a Fanconi-like renal syndrome in dogs following ingestion of large amounts of chicken jerky treat products [59,60]. Similar concerns about Fanconi syndrome in dogs have recently been raised in the UK [61,62].

Such incidents are relatively uncommon. However, commercial pet food brands may commonly be associated with a variety of hazards. These include significant quantities of abattoir products condemned as unfit for human consumption, such as “4-D” meat (from animals that are disabled, diseased, dying or dead on arrival at the slaughterhouse), labelled using terms such as “meat derivatives” or “by-products” [63,64]. Due to expensive labour costs, plastic ear tags are not always removed. Old or spoiled supermarket meat, sometimes without removal of styrofoam packaging (which increases labour costs), may also be used [65].

A variety of pharmacologically active compounds may result from residues derived from farm or industrial practices, some of which are illegal. The most devastating toxicity incidents have resulted from cross-contamination of feed ingredients with medicated feeds during feed or premix processing, handling or delivery. Significant cross-contamination of medicated residues in subsequent batches can occur even after multiple sweeper batches of un-medicated product have passed through the system. Ionophore antibiotics, for example, are included within commercially- available feed additives administered to poultry for control of coccidiosis, and to beef cattle and swine for improved feed efficiency and meat production. They include salinomycin, lasalocid, monensin sodium, and narasin, among others. However, they can be toxic to cardiac and skeletal muscles and peripheral nerves, and can cause paralysis and even death, in dogs and cats [58].

Fish are also commonly used within pet food. However, they have not evolved mechanisms to excrete modern oceanic pollutants, such as mercury and PCBs, which accumulate in their tissues, and can reach hazardous levels [27,66,67,68]. Due to the kind of bacteria and enzymes found in fish, and the effects of atmospheric oxygen, fish also decompose faster than terrestrial animals. The transportation chains are also lengthier, resulting in greater time periods for decomposition and putrefaction [69].

Additional potential hazards associated within commercial meat-based diets include free radicals, trans fatty acids, and other toxins from restaurant grease used as a fat source, hormonal residues, chemical preservatives, and the degradation of sensitive nutrients such as enzymes and vitamins—due to the temperatures, pressures and chemical treatments involved in processing. These have all been described in detail elsewhere [70].

### 6.2. Controlled Studies

Given the number of potentially hazardous ingredients found in commercial meat-based diets, it is not surprising that a significant number of controlled studies have demonstrated increased risks of a variety of diseases following long-term maintenance of cats and dogs on such diets, including kidney failure [71], liver, musculoskeletal, and neurologic diseases [72], birth defects [73], and bleeding disorders [74]. These have been described in more detail elsewhere [75].

As with the studies of vegetarian companion animals previously described, the standard of evidence offered by these studies does not always meet the standards of well-designed RCTs, considered the cornerstone of Evidence-Based Medicine [47]. Hence, similar caution must be exercised before drawing definitive conclusions from these results. Nevertheless, they do raise significant concerns.

In some cases, such studies have led to recognition of deficiencies which have since been rectified. In 1987, for example, Pion and colleagues [75] demonstrated low plasma taurine concentrations associated with echocardiographic evidence of myocardial (heart muscle) failure in 21 cats fed commercial meat-based cat foods. At that time, thousands of pet cats died annually from the heart muscle disease, dilated cardiomyopathy. Deficiency of the amino acid taurine may also result in retinal atrophy, causing visual deficits, developmental deficits of the visual cortex and cerebellum, reproductive failure, and thromboembolism. Normal growth, immune and neurological function are all dependent on adequate taurine levels [27,40,76,77,78,79]. Pion and colleagues demonstrated that oral supplementation with taurine reversed the disease, and, consequently, most meat-based and vegetarian pet foods are now supplemented with synthetic taurine.

In other cases, such deficiencies have not been rectified. The amino acid L-Carnitine, for example, is of potential importance to dogs at risk of dilated cardiomyopathy. This potentially fatal disease affects about 2% of all dogs, appearing mostly in large and giant breeds. A small percentage of these lack sufficient cardiac L-Carnitine, which is normally removed during processing, and not supplemented due to cost (Fortunately, alternative sources exist; e.g., www.Carnitine-taurine.com—see [26]) [80].

Additionally, as noted previously, Hill and colleagues [42] demonstrated significant disparities between labelling guarantees of nutritional content and actual nutritional contents of pet food brands in five of the United States. When using calculation methods recommended by the National Research Council, ME density was found to be significantly greater than advertised. Given that dietary variation is minimal for companion animals, when compared to human diets, and that chronic feeding is the norm, such an error constitutes a significant dietary hazard. It could partly explain the concerning rates of obesity and its sequelae in companion animals [43]. Within the UK, for example, a recent survey of 572 veterinary professionals reported that 81% believed they had observed increased pet obesity in the preceding two years, and 80% believed there would be more overweight than healthy pets within the next five years [81].

## 7. Natural Feeding Behaviour

A common concern about vegetarian companion animal diets is that they are “unnatural”. The concept of “naturalness” is increasingly important to consumers, although there is less clarity about what this actually means—with most having their own perceptions of what should be considered natural, based on personal experiences, biases, or preferences [13]. Nevertheless, there is a frequent presumption that domesticated cats and dogs should eat meat, because prey animals were consumed by their wild ancestors or counterparts.

It is illuminating, however, to compare the ingredients included within commercial meat-based diets with the nutritional needs and preferences of wild cats, dogs and wolves, and of domesticated cats and dogs. Such commercial diets commonly include body parts from cows, sheep, pigs, turkeys, ducks, chickens, fish and prawns; some of which have been labelled as unfit for human consumption. And cows’ milk is also commonly fed to cats, even though some (like humans) are lactose intolerant and, consequently, experience intestinal signs such as diarrhoea. In contrast, the natural diet of feral cats consists primarily of small mammals, birds, fish, reptiles, and invertebrates. Feral dogs are known to hunt in packs, similar to wild canines, and eat a wide variety of foods [20]. The diet of wolves consists primarily of animal protein typically sourced from larger prey, such as elk, with the nutrient-dense organs consumed first, followed by muscle tissue [82].

Clearly, there are significant differences between the prey species these animals would naturally consume, and the species routinely included within commercial meat-based diets. In addition, the latter can contain significant hazards, and a range of clearly unnatural ingredients. As described previously, these have included potentially pathogenic microorganisms, prion proteins, mycotoxins, antibiotic and hormonal residues, chemical contaminants such as melamine, plastic ear tags, styrofoam packaging, and heavy metal and organic pollutants within fish.

To encourage domesticated cats and dogs to eat such diets, dry food may be sprayed with a combination of refined animal fat, lard, used restaurant grease, and other oils that are sometimes considered too rancid or inedible for human consumption. These provide the distinctive smell that wafts from a newly-opened packet of kibble. However, deep frying of restaurant food results in rapid oxidation, producing free radicals, trans fatty acids, and other toxins. Repeated use of oil results in the build-up of such contaminants [64].

Digest may also be utilised. Digest is an industry euphemism for a soup of partially-dissolved intestines, livers, lungs and miscellaneous viscera of chickens (primarily) and other animals, produced using various enzymes and acids. The precise ingredients used are trade secrets, which, in differing combinations, produce varying flavours. Batches considered to taste more like beef can transform a can of miscellaneous body parts into “Beef Stew”, while those considered more “fishy” may result in “Ocean Whitefish” and so on. According to Lewis and colleagues [83] in *Small Animal Clinical Nutrition*, “Digest is probably the most important factor discovered in recent years for enhancing the palatability of dry food for cats and, to a lesser degree, dogs”.

When wild cats, dogs or wolves kill prey, they gorge as much as possible to prevent consumption by competitors. This is followed by uncertain periods of hunger. In stark contrast, domesticated cats and dogs are fed assorted body parts, usually from animals they would never naturally eat, dispensed from tins or packets at predictable times daily, with kibble sometimes available *ad libitum* (i.e., always). This bears little resemblance to natural feeding behaviour.

Companion animal owners frequently microchip, vaccinate, de-worm, de-flea and de-sex their animal companions, and confine them indoors at night because they correctly believe such steps are recommended to safeguard health. Clearly, such owners are willing to depart quite radically from “naturalness” when they believe it may protect the health and welfare of their pets. Accordingly, the resistance of such owners to the concept of vegetarian companion animal diets is more likely to stem from ignorance about the hazardous ingredients found within commercial meat-based diets, and about the potential of nutritionally-sound vegetarian diets to safeguard health, than from any deep-seated commitment to “naturalness”.

## 8. Safeguarding Health

Clearly vegetarian companion animal diets can avoid the hazards sometimes associated with meat-based diets. However, unless they are nutritionally complete and reasonably balanced, they will incur the hazard of malnutrition. To avoid this, owners can use a nutritionally-complete commercial diet, or can add a nutritional supplement appropriate to the species and life stage, to a homemade diet. Such supplements are available from commercial suppliers (e.g., Harbingers of a New Age—see [26]), along with recipes for formulation (and, in the case of kibble, baking).

As mentioned previously, dilated cardiomyopathy is a potentially fatal disease affecting about 2% of all dogs, appearing mostly in large and giant breeds. A small percentage of these lack sufficient cardiac L-Carnitine, which can predispose them to this disease. Hence, owners of animals at risk should consider appropriate supplementation (e.g., www.Carnitine-taurine.com—see [26]).

Most owners choose to feed commercially-available “complete” diets, which may offer advantages of convenience, cost, consistency, and potentially, better nutritional balance. As indicated by the studies previously reviewed, concerns exist about the nutritional soundness and reliability of a variety of diets, both vegetarian and meat-based. Owners should consider asking pet food companies what steps they take to ensure nutritional soundness and consistency of batches, and what, if any, evidence—particularly independent evidence—they can provide confirming nutritional soundness. If enough consumers made such inquiries, companies would probably respond by strengthening appropriate quality control standards, independent verification, and by publicising such information, all of which would increase consumer confidence and the safety and quality of diets for companion animals.

Particularly where grounds for such confidence do not exist (which presently remains the case with most brands, whether vegetarian or meat-based), owners should consider combining brands or gradually transitioning their pets onto different brands or diets every few months, in the hope that any deficiencies will at least differ between different diets.

Great patience and persistence may be required when altering diets, particularly for animals used to a particular flavour of digest. Changes are best made gradually, e.g., by feeding a 90%/10% old/new dietary mixture for a few days, then 80%/20%, and so on.

Gradual changes allow an appropriate transition of digestive enzymes and intestinal micro-organisms, minimizing adverse reactions such as abdominal discomfort, flatulence and diarrhoea.

Owners should clearly demonstrate that they consider the new diet is just as edible as the old (without possibly warning or alarming their pet by making a fuss). They should not be concerned if animals eat around new food at first. Simply having it in close proximity will help create the necessary mental association, as will mixing the food thoroughly. The addition of odiferous (the sense of smell is very important) and tasty additives, such as nutritional yeast, vegetable oil, nori flakes and spirulina, can all help, as well as gently warming the food. Offered food should always be fresh. Gradual change and persistence are the most important factors for transitioning resistant animals, however.

As with all companion animals, owners should monitor the health of their animals on a regular basis, including through regular checks of bodyweight, activity level and demeanour. Although checks should normally occur at least weekly, this should be an iterative process, with assessments as often as required. Factors to be evaluated should include the animal (e.g., high or low activity level, life-stage: <1 year or >7 years—gestating or lactating all warrant greater scrutiny), diet, feeding management and environmental factors [28]. Any problems, such as progressive weight loss, or more obvious signs of illness such as adverse coat changes, vomiting or diarrhoea, should trigger a veterinary examination; which should, in any event, occur at least annually. Owners should consider routine blood screenings (haematology and biochemistry) and urine tests during such wellness checks, and in the case of illness.

### Urinary Alkalinisation

For animals on vegetarian diets, one additional factor warrants consideration. The excretion of the nitrogenous waste products of protein catabolism results in the acidic urine of carnivores. Plants are relatively deficient in acidifying amino acids, and due to the higher pH of plant-based protein, vegan and vegetarian diets can result in more alkaline urine. The pH (acidity) alterations predispose to the crystallisation of certain urinary salts, resulting in the formation of stones in the urinary system (urolithiasi*s*), which may result in feline urological syndrome or canine equivalents: partial or complete urinary obstruction (which may be life threatening), dysuria (difficulty in urinating) and haematuria (blood in the urine) [78]. Struvite crystals (magnesium ammonium phosphate) are more likely to form in alkaline urine, and are of particular concern [50,84]. Although due to their narrow urethral diameters, male cats are most at risk, similar problems may occur in dogs, and in females of either species. Alterations in bacterial flora, with increases the possibility of urinary infections, may also result.

Accordingly, special attention to urinary pH is warranted for animals (and particularly, male cats) maintained on vegetarian diets. Regular monitoring of the urine acidity of both sexes of cats and dogs is essential, at least weekly during any dietary transition, illness or instability, and monthly after stabilization. Urine can be collected from dogs using containers such as foil baking trays, and from cats using non-absorbent plastic cat litter available from veterinarians. pH test strips are also available from veterinarians, although electric pH metres provide the most accurate results.

The normal pH of a cat’s urine is 5.5–7, and the normal range for a dog’s urine is pH 5–7 [85]. A pH > 7 indicates alkalinity. A variety of dietary products (e.g., “Vegeyeast” from Harbingers of a New Age—see [26]) and additives can correct alkalinization, should it occur. Asparagus, peas, brown rice, oats, lentils, corn, brussel sprouts and yeast may be included in feline and canine diets, and are all urinary acidifiers [27]. Vitamin C (ascorbic acid) is also a urinary acidifier. The British Small Animal Veterinary Association (BSAVA) *Small Animal Formulary* [86] recommends a dosage of 50–80 mg/kg every 24 h for cats and dogs. And for more serious cases, the amino acids methionine and cysteine may be used [13]. The BSAVA *Small Animal Formulary* [86] recommends a dosage of 200 mg/cat every 8 h. More detailed advice about urinary alkalinisation and corrective strategies is available via www.vegepets.info, or within veterinary medical texts.

Increased urinary acidity, decreased urinary magnesium and increased water consumption all help to keep the urinary pH within a healthy acidic range, and help to prevent the formation of struvite crystals. However, acidifying nutrients, agents, or products should be used carefully, as excessive levels can lead to metabolic acidosis. Increased urinary acidity may also promote higher urinary excretion of calcium and lower excretion of magnesium, and magnesium is a natural inhibitor to the formation of urinary stones associated with calcium [87].

## 9. Conclusions

As evidence accumulates about the links between degenerative health conditions, farm animal welfare problems, environmental degradation, climate change, and causative factors—such as animal farming and the consumption of animal products—consumer concerns about the adverse impacts of traditional meat-based diets are likely to increase. Accordingly, interest in alternative diets—including vegetarian diets—is likely to grow. It is entirely possible for companion animals to survive, and indeed thrive, on vegetarian diets. However, these must be nutritionally complete and reasonably balanced, and owners should regularly monitor urinary acidity and should correct for urinary alkalinisation through appropriate dietary additives, if it occurs.

Those interested in vegetarian companion animal diets should be aware of concerns about the nutritional adequacy of some such diets demonstrated by a number of studies over a significant number of years. However, to ensure a balanced view, they should also be aware that similar concerns exist about commercial meat-based diets.

They should also be aware that, although rarely conducted in accordance with the highest standards of evidence-based medicine, a significant and growing body of population studies and cases have indicated that cats and dogs maintained on vegetarian diets may be healthy—including those exercising at the highest levels—and indeed may experience a range of health benefits. Vegetarian animals also experience a range of health problems, but these problems are also prevalent in companion animals maintained on meat-based diets. Finally, pet owners should be aware that a significant body of additional studies have demonstrated health problems in domesticated animals maintained on various meat-based diets.

Regardless of dietary choice, consumers should be encouraged to check labelling claims of nutritional adequacy, and to ask manufacturers what steps they take, and what evidence they can provide, to ensure nutritional soundness and consistency of their diets.

And as with all companion animals, owners should monitor the health of their animals on a regular basis, including through regular checks of bodyweight, activity level and demeanour. Any significant, ongoing problems should trigger a veterinary examination, which should, in any event, occur at least annually, and biannually after approximately seven years of age. Biannual examinations are also advisable during the first year on a new, vegetarian diet. Owners should consider screening blood and urine tests during such wellness checks, and in the case of illness.

As interest in vegetarian companion animal diets continues to grow, it is anticipated that further relevant studies will shed additional light on the nutritional adequacy of these diets, and on the health of companion animals maintained on them. We intend to continue to provide summaries of any good quality evidence, at www.vegepets.info.
